# Cybersecurity, Data Privacy and Blockchain: A Review

**DOI:** 10.1007/s42979-022-01020-4

**Published:** 2022-01-12

**Authors:** Vinden Wylde, Nisha Rawindaran, John Lawrence, Rushil Balasubramanian, Edmond Prakash, Ambikesh Jayal, Imtiaz Khan, Chaminda Hewage, Jon Platts

**Affiliations:** 1 Cardiff School of Technologies, Cardiff Metropolitan University, CF5 2YB Cardiff, UK; 2 School of Information Systems and Technology, University of Canberra, Bruce, ACT 2617 Australia

**Keywords:** Cybersecurity, Data privacy, Blockchain, IoT, Smart Contracts, GDPR, ISO 27001

## Abstract

In this paper, we identify and review key challenges to bridge the knowledge-gap between SME’s, companies, organisations, businesses, government institutions and the general public in adopting, promoting and utilising Blockchain technology. The challenges indicated are Cybersecurity and Data privacy in this instance. Additional challenges are set out supported by literature, in researching data security management systems and legal frameworks to ascertaining the types and varieties of valid encryption, data acquisition, policy and outcomes under ISO 27001 and the General Data Protection Regulations. Blockchain, a revolutionary method of storage and immutability, provides a robust storage strategy, and when coupled with a Smart Contract, gives users the ability to form partnerships, share information and consent via a legally-based system of carrying out business transactions in a secure digital domain. Globally, ethical and legal challenges significantly differ; consent and trust in the public and private sectors in deploying such defensive data management strategies, is directly related to the accountability and transparency systems in place to deliver certainty and justice. Therefore, investment and research in these areas is crucial to establishing a dialogue between nations to include health, finance and market strategies that should encompass all levels of society. A framework is proposed with elements to include Big Data, Machine Learning and Visualisation methods and techniques. Through the literature we identify a system necessary in carrying out experiments to detect, capture, process and store data. This includes isolating packet data to inform levels of Cybersecurity and privacy-related activities, and ensuring transparency demonstrated in a secure, smart and effective manner.

## Introduction

With the recent emphasis on societies in increasing their dependency on cloud technologies, coupled with the human need to communicate and share data via digital networks, Internet of Things (IoT) devices to include smart phones, industrial and domestic appliances, continue to be a necessary function in conducting business. Social exchanges and transactional types of data for example, drive the financial markets thus facilitating in the swift development of emerging technologies at an ever faster rate to keep up with supply and demand trends. In a domestic setting, the sharing of digital media (videos, music, pictures, documents (data)) through messaging services to enhance subject areas such as information technology, sport, social sciences, education and health for example, IoT devices enable the efficient and effective transfer of data world-wide instantly via the Internet of Everything (IoE) via the cloud. In an industrial context, Smart Sensors, Application Programming Interfaces (API) and IoT networks facilitate remote working across digital boundaries globally.

These potentially devastating instances of data sharing and/or criminality, influence the confidentiality and protections set out by governments, businesses and organisations, culminating in legal and ethical disputes with significant financial ramifications due to Denial of Service (DDoS) attacks for example, that would damage and disrupt entire business data architectures, infrastructures networks and services on a large scale. Consequently, with society relying more and more on the exchange and processing of Personal Identifiable Information (PII) via IoT, trust in renowned institutions and government organisations to include broadcast and digital media outlets becomes a main issue. As a user chooses to share social network, personal and confidential information whilst shopping on-line for example, they should be aware of the nature and intent of cyber-criminality and have faith in the criminal justice system of a given territory.

On the other hand, for businesses, organisations, government bodies and academic institutions to be able to freely validate and authenticate their data in the service of societies globally, Artificial Intelligence (AI), Big Data (BD), Blockchain (BC) Combined Technologies and methodologies, contribute significantly in mitigating cyber-crime, whilst providing legal bodies the power to hold companies, organisation and institutions to account. One such method is the Smart Contract (SC) for example, and when utilised in the drafting and consenting of a legal document or digital certificate, provides an evidence-based transparent method in enhancing the legal credibility and value of a financial transaction. As a function of BC, the SC is validated, implemented then shared across a Pier-to-Pier (P2P) network as a Distributed Ledger Technology (DLT) for all parties to see which provides transparency and accountability.

### Cybersecurity

When utilising elements of cybersecurity, these technical requirements facilitate in the effective management of IoT hardware and software operations, physical interfaces and internal policy development. Additionally, the management system ISO 27001 supports network communication protocols, data access control and cryptography (i.e., password encryption), that contribute in ensuring a robust and secure communication method inclusive of cybersecurity staff training; all whilst minimising network communication attacks in the presence of malicious third-parties [1].

However, to harness and derive value from the volume, variety and veracity of data available, concepts such as BD, AI and Machine Learning (ML) utilise prescribed algorithms and analysis techniques across vast quantities of public, private and sensitive data through digital networks, that exponentially increases the risk of data breaches, viruses and malicious attacks. In other words, in successfully utilising these technologies in the legal acquisition and processing of data from the public and private sectors, also to include practical user measures, potentially reveals challenges and vulnerabilities that can further expose a user or group to cyber-criminality.

### Data Privacy

Additionally, the ISO 27001 framework functions in conjunction with the General Data Protection Regulation (GDPR) Regulation (EU) 2016/679, and Data Protection Act 2018 c. 12 (DPA), in facilitating personal data controls and measures in the UK and European Unions (EU) digital boundaries. In processing medical data for example, a mandatory Data Protection Impact Assessment (DPIA) is undertaken in identifying and establishing the risks alongside eight core principles to include; lawful and ethical methods of data acquisition, data storage security and duration, fair use, and for data to be kept within specified locations and regions [2].

In utilising these legal frameworks and management systems, tracking tools such as ‘cookies’ for example may utilise the aforementioned AI, ML and algorithmic analysis unlawfully, and as a result, a user may not be aware of the tracking nature and capabilities contained within the software for analysis and marketing purposes. Additionally, without user consent, the awareness and continual levels of maintenance required of said cookies, that are a necessary function in surfing the web, could expose business networks to anti-forensic methods, legal jurisdiction matters, system hardware and Service Level Agreement (SLA) breaches, which compound over time and further aggravate technical, legal and ethical challenges in operating IoT devices in a compliant, safe and secure business environment.

Furthermore, when utilising in a healthcare service context, a SC policy with cryptography as a cybersecurity control method, gives transparency, protected agency and responsibility to the public, financial markets, business professionals and legal representatives, in conducting valid and transparent actions or investigations on behalf of the directorate or client. When this method is applied retrospectively, it also gives accountability in upholding vigilance and resilience when managing cyberspace, an operators duty of care and consideration of confidential data breaches, its sharing, and ramifications of exposing vast amounts of confidential National Health Service (NHS) patient data for example [3].

### Blockchain Security

BC based functions, methods and systems utilise concepts like Cryptocurrency (i.e., Bitcoin and Etherium) as an alternative to fiat currencies, representative consensus protocols, anonymous signatures, off-chain storage and non-interactive zero-knowledge proofs. These concepts provide validity, anonymity, and transparency when coupled with inner corporate or organisational audit, policy deployment, healthcare provider and security service function of carrying out legal and domestic activities. This system is trustless by design and offers promise for equitable and transparent transactions.

## Overview

**Fig. 1:**
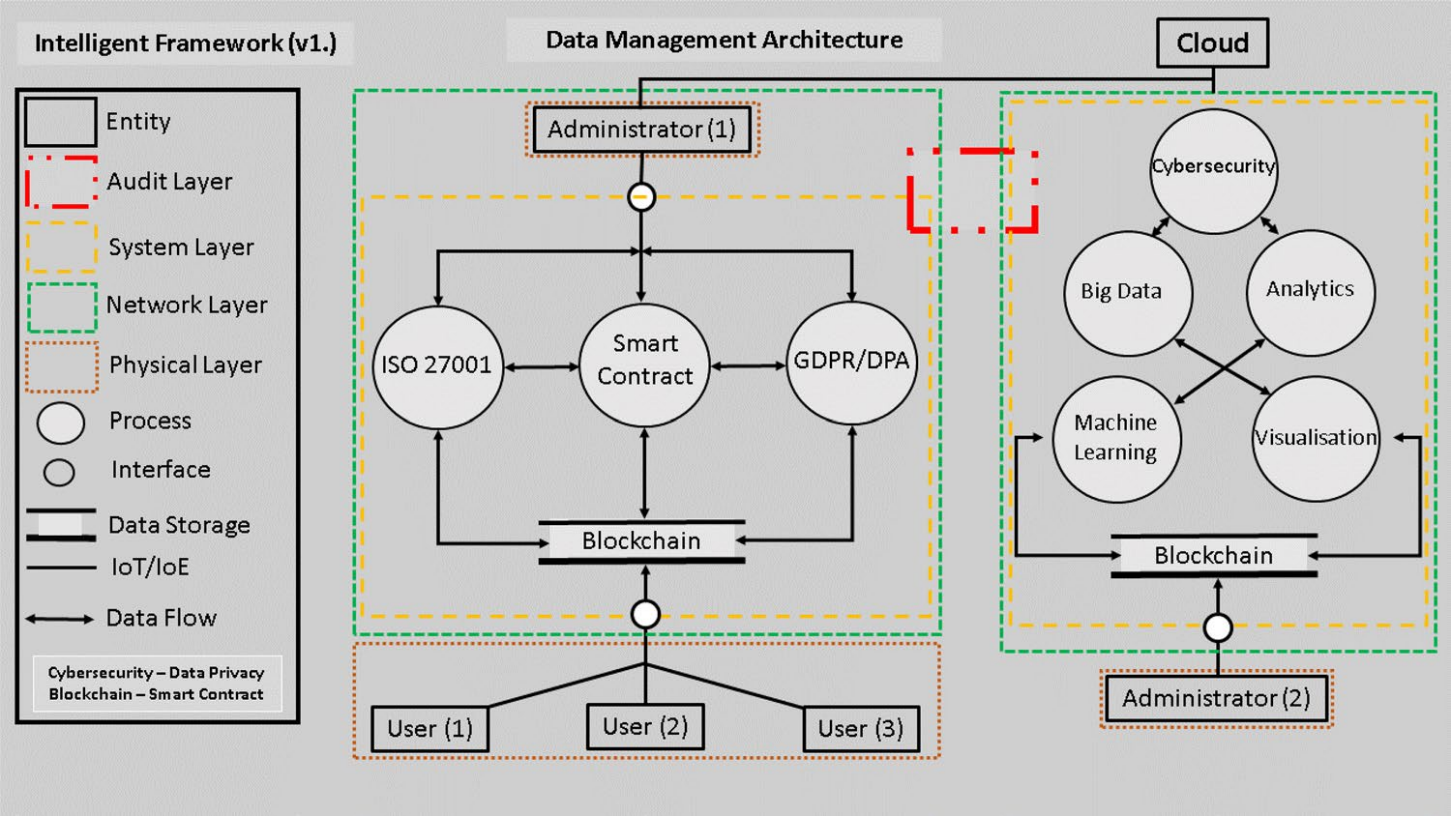
Data flow audit mechanism

As per all the above, this review and study proposes an intelligent framework to aide in the identification and detection of compromised network packet data. The use of BC and SC are to be utilised as an information carrier (data) and for evaluation, validation and testing with pre-prescribed control protocols. Then, to conduct a literature review in ascertaining current methodologies, techniques and protocols in aiding the development of said framework. To minimise human intervention, an intelligent automated approach is utilised in the capturing of network data at pre-determined intervals. Ultimately, the data events are tested against a framework with analysis of findings to demonstrate comprehensive framework feasibility (see Fig. 1).

## Cybersecurity

Cybersecurity refers to: *“a measure for protecting computer systems, networks, and information from disruption or unauthorized access, use, disclosure, modification or destruction”* [4]. Therefore, in trying to understand cybersecurity and its applications towards IoT and smart devices, brings additional questions that need analysis through various notions of cyberspace. One solution is unifying all the terminologies above to bring together the importance of understanding where network intrusion comes from, how it is detected, and how prevention of cyber threats occur. When looking at prevention, AI and ML uses could also potentially contribute to the rise in using this technology to secure and protect data [5].

### Cybersecurity IoT and ML

As Information Technology (IT) facilities expanded, overall digital technology saw growth in more devices being introduced and connected to the internet, so that access to data is freely available to allow for more activities to be undertaken. These activities allow for outcomes to be predicted [6]. Therefore, in response, various ML mathematical algorithms allow for classification usage such as Support Vector Machines (SVM), Decision Trees and Neural Networks. These algorithms all compound and highlight how data is treated and managed to produce an outcome, and predictability that is required to contribute to economic growth as societies move forward. ML capabilities go far beyond the expectations of conquering human hobbies, but lends further into everyday chores and events in daily lives.

Other real-life examples of ML usage rest in many industries focusing on identifying fake news, implementation of spam filters, identifying fraudulent or criminal activities online, and improving marketing campaigns. These large quantities of data are often private and sensitive, whilst travelling through Cyberspace transferring data along the way. Disadvantageously, this existence of cyberspace creates a wider security attack surface for potential malicious activities to occur. This demonstrates that human factors and the large influence it has on the security of IoT [7] is highly impactful.

Humans’ perceptions of security and privacy concerning these devices are also a subject to be discussed, for example, the concept of ‘Cookies’ as a tracking tool for online web surfing, and its safety measures, which are often shoehorned as a debate in itself, and the awareness of how it should be used has been seen through glazed eyes [8]. However, recent reports suggest that many contributory questions arise from understanding IoT and the safety net around it, and how humans cope and live alongside IoT. Anti-forensic methods, jurisdiction and Service Level Agreements (SLA) for example, all further aggravate technical, privacy, security, and legal challenges. In addition, the presence of GDPR and IoT, coupled with the human factors involved, present immense challenges in keeping these devices safe and secure.

### Cybersecurity and SMEs

UK Small to Medium Enterprises (SME’s) have always seen challenges in understanding cybersecurity due to the increase in threats that have risen in recent years. The European Commission’s employment criterion for an SME minimum cyber-criterion is that for any business that employs less than 250 people [9]. The challenges faced are both operational and commercial in SMEs using Intrusion Detection mechanisms coupled together with AI and ML techniques in the protection of their data.

SMEs intrusion, detection, and prevention methods has become a priority in the realisation of keeping their data secure and safe with the integration of real-world objects and IoT, with understanding how ML techniques and AI can help secure zero-day attacks. Rawindaran et al. [1] took particular interest in the SME market and showcased an experimental scenario in which the intrusion, detection and prevention models were compared, and the views of the SME examined. The study looked at the various approaches in identifying ways to detect and protect any intrusions coming into the network and what operating devices would help in this process. The paper also explored the understanding in trying to protect the data and how government policies and procedures such as GDPR in the UK/EU, could assist towards this process [10].

### Cybersecurity and SME Attacks

Rawindaran et al. [11] further examined the impact of how threat levels of attacks such as Ransomware, Phishing, Malware, and Social-engineering amongst others, were compared between an Open-Source device, such as SNORT and pfSense, and Commercial Network Intrusion Detection (NIDs) such as Cisco. There were three different NIDs and their features were compared. It was concluded that whilst SNORT and pfSense were free to use from the Open-Source market, it required a certain level of expertise to implement and embed the rules into a business solution. It was also noted that Cisco, due to their engineering expertise and their position as market leaders in the industry, were able to embed these free rules and use it to their advantage.

What emerged from this study was how businesses and organisations with the help of government policies and processes, needed to work together to combat these hackers, malicious actors, and their bots, and manage and stay ahead of the game [4]. The paper also discussed various ML approaches such as signature based models and anomaly based rules used by these devices to combat these attacks [12].

Additionally, signature based models could only detect attacks that were known, whereas anomaly-based systems were able to detect unknown attacks [13]. Anomaly-based NIDs made it possible to detect attacks whose signatures were not included in rule files. Unfortunately, due to the maturity of Anomaly NIDs, the costs were still very high to run and required computing power that were unrealistic in the SME environment. Anomaly based NIDs whilst still in its infancy, require a deeper analysis and future study.

Rawindaran’s study provided perspectives on better comparisons and relative conclusions and how it was important to explore further both the empirical as well as in scenario analysis for different dimensions, the nature and context of cyber security in the current world of internet and cyber connections. Rawindaran also explored how ML techniques have become vital in the growth and dependencies of these SMEs in the UK in their operations and commercial environment. This study took on an initial look at success stories from big technology companies such as Amazon, Google, and Facebook, in their use of ML techniques for their cybersecurity [14]. The methodology adopted in this study focused on structured survey questions on a selected sample number of respondents and directed its questions to the SMEs management, technical and non-technical professionals.

### Cybersecurity and ML to Mitigate Attacks

Rawindaran et al., found that awareness of ML and its uses is still on a learning curve and has yet to be defined. The study brought to surface the three main categories of ML that being Supervised Learning, Unsupervised Learning and Reinforcement Learning and the algorithms that sit behind them [15]. Examples of Supervised Learning included real life predictive text in tweets in Twitter and product reviews in Amazon and eBay, calculating temperature, insurance premiums, pricing, and number of workers to the revenue of a business.

Examples of Unsupervised Learning include examples include identifying fake news, implementation of spam filter, identifying fraudulent or criminal activity online, and marketing campaigns. Reinforcement Learning shows example of playing a video game that provides a reward system when the algorithm takes an action. Each learning method used algorithms that helped with calculations and predictions and a dataset that helped in the development and structures of its uses. It also deducted and quantified examples and showed strength in the SMEs perception and awareness towards ML and its uses.

The methods of ML and its algorithms lead into the focus of this study in which SMEs were given the opportunity to make themselves aware of these algorithms that exist within their own cybersecurity software package. Further the analysis of this study showed the existence of these algorithms such as Neural Networks, Support Vector Machines, Deep Networks and Bayesian, however most of these were cleverly embedded within the software used [16].

The initial idea of using an Intrusion, Detection and Prevention System (IDPS) method, from either a commercial or Open-Source device to protect the data of the SME, comes with the knowledge of ML and AI. As hackers become increasingly clever and the uses of bots take over, their ‘attacking’ methods, as protectors of the systems, society has had to lean on ML and AI technology to help. An IDPS system is able to help through the use of ML, to learn about malicious patterns compared to valid patterns on the internet. These various approaches are needed to protect and shield data. ML through anomaly detection, proved to be more effective in its zero-day detection than that of signature based in its effectiveness towards cybersecurity and adoption within the UK SMEs. There is a significant gap that needs to be fulfilled by perhaps more variations in the devices used for SMEs such as opensource and voluntary participants from knowledge of the community to keep future proofing these devices.

### Cybersecurity and Adversarial ML

With the increased use of ML in Intrusion Detection Systems (IDS) and IDPS systems within cyber security packages of SME communities, there suddenly lies the introduction of a new type of attack called Adversarial Machine Learning (AML) [1]. In a paper by Anthi et al. [17] states that with the introduction of ML IDSs, comes the creation of additional attack vectors specifically trying to break the ML algorithms and causing a bypass to these IDS and IDPS systems. This causes the learning models of ML algorithms subject to cyber-attacks, often referred to as AML.

These AMLs are thought to be detrimental as they can cause further delayed attack detection which could result in infrastructure damages, financial loss, and even loss of life. As [17] suggests, the emergence of Industrial Control Systems (ICS) plays a critical part on national infrastructure such as manufacturing, power/smart grids, water treatment plants, gas and oil refineries, and health-care. With ICS becoming more integrated and connected to the internet, the degree of remote access and monitoring functionalities increases thus becoming a vulnerable point target for cyber war. Additionally, with ICS more prone to targeted attacks, new IDS systems have been used to cater for the niche market of ICS, thus introducing vulnerabilities in particular to the training model of ML.

With the introduction of these new IDSs, has also introduced new attack vectors into the mix. The definition of AML provided by Anthi states that: “The act of deploying attacks towards machine learning-based systems is known as Adversarial Machine Learning (AML) and its aim is to exploit the weaknesses of the pre-trained model which has ’blind spots’ between data points it has seen during training”.

This is challenging as ML usage in IDS is becoming a tool used in daily attack detection. The study showed how AML is used to target supervised models by generating adversarial samples and exploring and penetrating classification behaviours. This was utilised by the use of authentic power system datasets to train and test supervised machine learning classifiers through its vulnerabilities. The two popular methods that were used in AML testing were automatically generated perturbed samples that were the Fast Gradient Sign Method (FGSM) and the Jacobian based Saliency Map Attack (JSMA).

Both methods showed how AML was used in penetration of systems through ML training models leading onto cyber-attacks. In another study by Catak et al. [18], further explored the security problems associated with AML, this time through the networks of 6G applications in communicative technology, that focused on deep learning methods and training. With the rapid development and growth of deep learning and its algorithms in the future technology pipeline of 6G was to further understand the security concerns around it.

Cataks’ paper [18] produced faulty results through manipulation of deep learning models for 6G applications to understand AML attacks using Millimetre Wave (mmWave) beam prediction in this case. AML mitigation and preventative methods were also used to try and stop these attacks from occurring for 6G security in mmWave beam prediction application with fast gradient sign method attack. In conclusion to Cataks’ paper found that several iterations of introducing faulty results gave a more secure outcome of the performance and security of the device. ML deep learning methods and algorithms were able to use these faulty results in altering the adversarial training approach. This increased the RF beam-forming prediction performance and created a more accurate predictor in identifying these attacks against the ML applications use.

## Cybersecurity: Summary

As with any new technology that stems to improve the cyber highways in lessening the effects of cyber-attacks, it is always coupled by the counterattack measure within this space. Being aware of these adversaries and future research will help reduce, or at least control the level of attacks being present in any cyberspace and landscape moving forward. The recognition of funding gaps that could be fulfilled by the government to support SMEs in the form of grants, subsidies, and similar financial assistance, through various public sector policies is also an important route to consider. Awareness and training for all SME management and their staff is important to understand the basic and perhaps advanced appreciation of cybersecurity through the eyes of ML and AI.

Whilst technology giants might lead the path in its implementation of ML and cybersecurity through its many variations of intrusion, detection, and prevention methods, it is these firms that will set precedence and bring awareness down to a SME level and the importance of ML in keeping our cyber world safe. Understanding whilst ML is increasing in usage through IDS and IDPS systems to reduce the cyber attack footprint, means that the rise in AML also is something to be concerned about.

## Data Privacy

An example in GDPR Recital 4 and in the proceeding Directive 1995/46/EC Recital 2, a main objective “the processing of personal data should be designed to serve mankind”. For this purpose, the Data Controller ensures legal compliance and legal justification of data processing out of necessity (not only processing convenience) and proportionality. For the acquisition of high-risk health data for example, GDPR mandates that a DPIA is carried out to mitigate risk and assess risk level to include if the data should be processed or not [19]. With data protection law, the UK and EU demonstrate cooperation, ethics, transparency with robust control methods in mitigating data privacy breaches. However, this also brings attention to the range of legal frameworks and the general movement of people globally. This should inform governments and business in data protection strategies.

### Data Privacy: Legal Frameworks [UK-EU]

Between the UK and EU, the Data Protection Act 2018 (DPA) and General Data Protection Regulations 2016 (GDPR) function together in overseeing how businesses, organisations and governments, utilise personal data. Eight key objectives guide anyone responsible for the handing and processing of personal data, and strictly imposes that data has to be lawful [acquisition], fair, accurate and up-to-date, not kept longer than needed, kept safe and secure, and not to be transferred outside the European Economic Area (EEA). By design, GDPR encompasses human rights with additional data collecting and processing principles (e.g. purpose, data-types and processing duration) [20].

### Data Privacy: SARS-Cov-2: Covid-19

In supporting the effort in mitigating disease transmission from the coronavirus pandemic (Covid-19), the cloud, cell-networks and IoT devices such as smart-phones, sensors and domestic appliances, continue to play a vital role in a wide range of global Tracing-Testing-Tracking programs. Many different approaches are adopted by global communities in minimising person-to-person transmission [21, 22]. This demonstrates that in response to the pandemic, coupled with the urgency in developing and deploying digital solutions, data privacy implications become ever more challenging with increasing data privacy risks. As a result, the handling of personal data [acquisition] research has developed and expanded [23].

However, in mitigating data privacy risks under adverse social and environmental conditions, it is not simply a matter of deploying digital solutions. The challenges presented in terms of service delivery (consistency, proportionality and transparency), also potentially increases the risk of data privacy breaches. Therefore, in terms of scalability via the cloud, partnerships between populations, businesses and governments could harmonise policy development and implementation with digital solutions.

### Data Privacy: Consent—Contact Tracing Apps

In a Republic of Ireland survey conducted with over 8000 participants, it was found that 54% would accept using a contact tracing app. Similarly, in the UK from a survey of 2000 participants found that 55% would accept using a government-controlled app, with higher uptake specifically for the NHS contact tracing app [21]. This information demonstrates a lack of app uptake in the remaining 45% of the British population that could undermine a governments ability in effectively handling data collection and the processing of critical medical information.

In contrast, other countries infer citizen consent when data collection is initiated for the public good. Meaning that private parties’ access to data is also endorsed by governments. Amnesty International (2020) also brings attention to many instances of questionable data privacy practices throughout numerous countries [21]. The examples potentially show the scale of data protection perceptions and attitudes and how they are interpreted, thus justifying a more focused and intensive approach to data privacy collaborative research. By analysing a variety of legal and regulatory frameworks, solutions and practices in a pandemic or crisis situation, we can learn how to effectively apply powerful and scalable outcomes. For example, robust and transparent data is necessary for the urgently needed Covid-19 vaccine distribution efforts for each nation [24].

### Transparency: NHS Test-Trace App

In response to the pandemic, the UK Government and NHS X (Digital) contact tracing app, aided by the private sector, brought into question their overall GDPR utility and compliance. Sub-contractors and companies that represent NHS X are also considered as processors of data, which bring additional GDPR compliance pressures. In this instance, the NHS X app code and DPIA was voluntarily submitted to the Information Commissioners Office (ICO) without the data store. This potentially highlights a lack of transparency with GDPR compliance, health surveillance capabilities and data storage capacities. The Joint Committee on Human Rights (JCHR) for example, were concerned at the rapid development and deployment of the contact tracing app in March 2020 [19].

### Data Storage and Identification

Clear definitions and solutions are needed for data and storage methods. Currently, obtaining an integrated and comprehensive view of (1) internal organisational personal data storage, (2) full organisational content comprehension of regulation, and (3) an auditable trail of necessary data processing activities [20]. Although GDPR compliance has significantly enhanced personal data protection (e.g. PII, PII sharing via add and marketing, collecting and sharing location data, child PII sharing, law enforcement, and data aggregation), more research is needed in facilitating a users right to erasure, to update and delete data and to completely satisfy the GDPR promise [25].

### Accountability and Traceability: BC & SC

To aide government transparency and societal trust, part of a solution is robust data privacy and accountability policies. Antal et al., discusses how BC can be effective in traceability, transparency, vaccine ID, assurances of it’s delivery, storage to include self-reporting of side effects. The authors implement a BC strategy using the inherent integrity and immutability of BC with ’in case of beneficiary registration for vaccination’ provision, thus eliminating identity impersonations and identity theft [26].

An example from Honduras demonstrates how a Toronto-based technology launched ’Civitas’, with user and government linked ID on a BC-based network. The BC contains the necessary data for determining when an individual can buy medicine, go food shopping, and also data to inform government agencies in resource and deployment strategies [27]. The GDPR for example, would conflict with this contact tracing methodology. More specifically, the right for a user to be forgotten (Article 17: Right to Erasure) due to BC immutability, and processing speed that would also inhibit BC network uptake and scalability.

However, BC in this case could operate within the confines of management and governance of BD repositories and warehouses whilst leveraging SC to enhance accountability, transparency and consistency in the appropriate forum.

### Trust: Vaccine Hesitancy in UK Households

Whilst a global effort was underway in mass vaccination programs, the UK strategy highlighted disparities from a lack of public engagement between public health bodies and ethnic minorities from historic mistrust and a lack of understanding in technology [24, 28]. Additional hesitancy included acute and chronic health effects from the vaccine.

A UK survey from 2020 for example, illustrated how Black, Asian, Minorities and Ethnic (BAME) communities had high vaccine hesitancy rates, when compared to white ethnic populations [28]. In Robertson 2021, the authors state that *“Herd immunity may be achievable through vaccination in the UK but a focus on specific ethnic minority and socioeconomic groups is needed to ensure an equitable vaccination program”* [29]. Including a more targeted approach to mental illness and disability [30].

## Data Privacy—Summary

In a global setting, is it possible to ethically and accurately collect data [also without consent] whilst also providing legibility for effective data collection, resource allocation and deployment strategies? A small part of the solution is in gaining a populations’ trust in technologies such as NHS app uptake, and for future research in global deployment strategies. This means a wide-ranging and continual assessment of legal frameworks and outcomes between companies, organisations and institutions for long-term data privacy planning. Strategies also include ensuring groups and individuals have faith in their data integrity in the cloud.

As necessary components of GDPR, the collecting, processing and deleting data remain a challenge. The enable user to fully engage with confidence, education and engagement with minorities, and with mental illnesses is an effective way to provide group assurances. As with different countries, data protection concepts and public engagement practices vary significantly. For anticipating any future disaster or pandemic scenario, it is clear that accountability through public engagement should help restore national and international trust. Also research needs to be undertaken to design and promote a flexible and global strategy to encompass technical solutions, operational resource strategy, and policy development. This would enhance data protection objectives, build population trust in government monitored apps and ultimately provide a successful and robust global protection strategy.

## Blockchain for Security

### Blockchain—Integrity of Data

BC is one of the most commonly discussed DLT for ensuring the integrity of data storage and exchange in trust-less and distributed environments. It is a P2P decentralized distributed ledger [31] that can enable trusted data exchanges among untrusted participants in a network. BC systems such as Ethereum and Hyperledger fabric, have become popular BC frameworks for many BC-based software applications. Core features of BC such as immutability and decentralization are recognized by many sectors such as healthcare and finance to improve their operations. Although BC is a relatively new technology—just over a decade old—it seems to be revolutionary and there is a substantial number of research articles and white papers to justify this remark.

### Blockchain—Cybersecurity

It is important to answer how emerging technologies such as BC can offer solutions to mitigate emerging cybersecurity threats and there is great research interest to study how BC can provide foundations for robust internet security infrastructures [32]. Many of the articles propose frameworks, prototypes and experimental beta BC-based solutions to problems in complex computing systems. Most of these experimental solutions are developed on Ethereum and Hyperledger fabric. In the case of Hyperledger fabric for example, this is due to its ease of software development, extensive customisability and interactivity.

Although Bitcoin is a most popular BC network, it has many cons such as its latency and great resource requirement. Some of practical solutions among them use innovative techniques to resolve critical cybersecurity issues. However, they imply infeasible changes to the existing system infrastructures that are difficult to readily test for efficiency and effectiveness when compared with conventional cybersecurity frameworks [33].

### Blockchain—IoT

In our increasingly interconnected IoT world, there is a great need to improve cybersecurity. As explained in [34, 35], cyber-attacks that exploit vulnerabilities in IoT devices raise serious concern and demand for appropriate mitigation strategies to tackle these threats. Ensuring integrity of data management and malware detection/prevention is an exciting topic of research [36].

It should be noted here that BC cannot eliminate cyber risks, but it can significantly minimize cyber threats with its core features. While most IT systems are built with cybersecurity frameworks that use advanced cryptographic techniques, they rely on centralized third-party intermediaries such as certificate authorities to ensure the integrity of their data management. Malicious parties can exploit weaknesses in such relationships to disrupt/penetrate these systems with cyber threats such as DDoS attack, malware, ransomware, etc.

### Blockchain—Protocols

BC can resolve these issues due to its decentralization; it eliminates single points of failures and the need for third-party intermediaries in IT systems and ensures the integrity of data storage and exchange with encryption and hash functions [37] so that data owners can completely audit their data in the systems.

A BC network with many mutually trustless nodes is more secure than a network with few nodes that rely on trusted/semi-trusted centralized third-party intermediaries because, in a BC network, every node has a complete copy of the unique record of all transactions in the network that is maintained with the network consensus protocol. The robustness of a BC network i.e. its safety and security, depends on its decentralization, and this depends on its governance and consensus protocols. A good comparative study of DLT consensus protocols is provided by Shahaab et al. [38].

### Blockchain—Summary

What are some future research directions and challenges for BC and Cybersecurity?

1. Consensus Protocols: Generally, public BC networks have high latency due to their consensus protocols. This makes them a non-starter for applications in real-time environment. Research on consensus protocols should be holistic and consider both, hardware and software, for such environments [39].
2. Cryptocurrencies: more research on cryptoassets is needed to tackle challenges to legal enforcement and forensics - both domestic and international—that enable cybercriminal activity such as terrorism financing.
3. IoT: As explained in [40], consortium BC networks can be used to improve the overall internet connectivity and access. Future research on IoT-BC integration should demonstrate feasible implementations that can be evaluated and compared with existing IoT solutions. They should also quantitatively study fault tolerance, latency, efficiency, etc. of BC-based IoT networks.
4. Data Analytics: BC can ensure the integrity of data and with AI/BD analytics it can be used to reduce risks and fraudulent activities in B2B networks. Hyperledger fabric is a DLT project that can be used for this relatively unexplored research areas.

## Cybersecurity, Data Privacy and Blockchain

As stated in [41], BC-based digital services offer transparency, accountability and trust, however not one size fits all, as there are paradoxes between cybersecurity, GDPR compliance and the operation of BC. Haque et al., demonstrate in a systematic literature review regarding GDPR-BC compliance and highlights six major categories that are:

1. Data modification and deletion (Articles 16–18)
2. Default protection by design (Article 25)
3. Controllers/processors responsibilities (Articles 24, 26 and 28)
4. Consent management (Article 7)
5. Lawfulness and principles (Articles 5, 6 and 12)
6. Territorial scope (Article 3)

Haque et al. [41] states that use-cases of BC should be retrospectively applied in a way that can be made compliant to GDPR. The literature review also highlighted additional GDPR-BC research domains that include areas such as smart cities, information governance, healthcare, financial data and personal identity.

### GDPR vs Blockchain

Vast amounts of PII are being collected, screened, and utilsed illegally due to cyber-espionage, phishing, spamming identity theft, and malpractice. BC on the other hand, due to the immutability in design and utility in tracking, storing and distributing DLT data, can clash with GDPR, especially with the “Right to be forgotten: Article 17”, including various rights to erasure [42]. Al-Zaben et al., proposes a framework that is on a separate off-chain mechanism that stores PII and non-PII in a different location. It is best to design and regulate network participation in fulfilling GDPR requirements, although not a perfect fit, this example shows how by design, a compliant use-case can be augmented in fulfilling parts of GDPR.

### Ransomware Defense vs Blockchain

In [43], their paper describes that for malicious software to use configuration commands or information, malware has to be able to connect to the original owner. Therefore, a fairly new principle of domain generation is proposed, in that actively deployed ransomware is utilised to track user coordinates based on transactional data in a bitcoin BC. The gives a malware author the ability to dynamically change and update locations of servers in realtime.

### Supply Chain Attack vs Blockchain

Recent and alarming increases in supply chain cyber attacks, has given various implementation strategies of BC in security of IoT data, that generally produces positive outcomes due to the transparency and traceability elements inherent in the technology by design. This paper highlights and discusses challenges to include many BC based systems in various industries, and focuses on the pharmaceutical supply chain. In conclusion, [44] states that the application of BCT can enhance supply chain security via authenticity and confidentiality principles.

### Data Storage vs Blockchain

Due to the full-replication data storage mechanism in existing BC technologies, this produces scalability problems due to copying at each node, thus increases overall storage per-block [45]. Additionally, this mechanism can limit throughput in a permissioned BC. A novel storage system is proposed to enhance scalability by integrating erasure coding that can reduce data acquisition per block and enlarge overall storage capacity.

### Summary

Of the many challenges that face legal, operational and performance criteria with utilising BC, it is clear to see that as we gather more and more personal data, endure more cyber attacks, and encounter storage disadvantages, many proposed frameworks seek to provide solutions that are only a part of compounding and escalating situation. The transactional speed and scalability of technologies such as BC, can hinder data protection rights, focused cyber-attacks, and the ability to update and track users, however there are advantages in creating separate mechanisms that when produced as a whole, that can indeed support data verification, transparency and accountability in many industries.

## Results: Brief Overview of Intelligent Framework

Key Data Management Architecture Components: Fig. 1 shows the block diagram of the proposed framework. Key components of the framework are explained and synthesised in the following paragraphs.

### Blockchain: Data Storage and Immutability

To provide system accountability, transparency and traceability from network system traffic point of view, an article by Kumar et al., 2020 demonstrates how DLT systems are applied in e-commerce to include health medicines, security devices, food products to ensure BC technological and e-commerce sustainability. Also, [46] presents a study that explores the potential of DLT in the publication industry and present a technological review. The studies demonstrate how research is being explored and influencing DLTs globally alongside their synergies of application across academic, private and public sectors.

### Standardisation of IoT Interface Portal

For purposes of legal acquisition and processing of data with consent, users can connect from IoT smart devices and appliances, such as; smart phones, sensors, tablets and user desktops. User applications and interfaces also provide a level of protection by design in most cases, however the applications can also compound and conflict with each other to produce security vulnerabilities (e.g. Cookies). Networks include; Cellular, Local and Personal Area Networks (PAN/LAN), Low Power Wide Area Networks (LPWAN) and Campus Area Network (CAN) carrier methods operate and maintain IoT system stability. Some IoT devices are capable of ensuring seamless connectivity in data access. However, at the point of access, a user interfaces with a given IoT device could be one of multiple architectures that present challenges in correctly identifying and processing data in a legal, reliable and consistent fashion. Therefore an overarching framework to ensure a standardised system whilst mitigating risk (security Vulnerabilities) is catered for in utilising network protocols with a prescribed profile limited to key information such as, Personal Identification Number (PIN), Account Number and password encryption.

### Administrator 1: Public LAN/WLAN/CAN

A main purpose here is the execution of network communication protocols for the processing and or keeping (storage) of PII and data access control to include cryptography. At the level of an SME, the types of regulatory compliance’s necessary to operate as a business include a retrospective and current auditable trail to demonstrate good practices. A selection of operational scenarios are to be emulated (e.g. from case law) in the preparation of codifying, selecting and the setting of chosen principles, standards and legal frameworks. Other objectives to explore include, Confidentiality, Integrity, Availability and Data Minimisation. As shown in [47], stakeholders are required to initialise and validate a product block, this activates the wallet, to include pseudo-identity generation with a public and private key pair. The keys are utilised for signature and verification processes. Here, administrator 1 oversees and combines the execution of network communication policies to govern a user or a given set of protocols.

### Administrator 2: Private LAN Network

The function of the administrator here is to utilise criteria to facilitate accountability, transparency and traceability from network system traffic. Data entry points provide group integrity as each user, or entry, is available for all to see. More fundamentally, this data will help inform, develop, calibrate and test the setting of audit and assessment parameters. The information is then combined, contrasted and compared to the Administrator 1 data collection. Resulting information then updates the Valid Data Acquisition IDPS System and Cyber-Detection Methods (e.g. Packet Sniffing) of Network Packet Data communication protocols with data effective access control. In this case, Administrator 2 provides an array of users insights into the performance of ISO 27001 and DPA/GDPR policies to identify optimum operational cost in various prescribed operating scenarios. Through analysis with tools such as BD Analytics and ML for example, nuanced data, pattern identification and aggregation provides a basis for speculation as to an ideal operating system from within a business.

### Smart Contract: Agreement or Terms of Contract

Unfortunately, maintaining these systems incur at significant cost, on the other hand, these systems also cut out the “middle-man” and save resources to empower individuals and business owners. For example, individual and group scenarios are negotiated and interpreted between users in partnerships. In emulating this function, key objectives are identified and embedded from legal frameworks to produce an automatic transaction protocol with consensus in the implementation of a codex (e.g. OPCODES). Therefore, a codex of legal precedent and statutory instrumental data protection, data operation and dissemination laws will be emulated to start. The codex is the library and framework that enables partners to equitably participate in a sustainable and trust-less operational environment. In utilising ISO 27001 for example, a collection of policies are negotiated and agreed upon prior to formally undertaking a contract between parties. Therefore, GDPR and ISO 27001 are transcribed, layered and mapped with verification mechanisms derived from case-law and by design into a SC agreement. This dynamic process forms the centre of any given exchange or process of data acquisition and data dissemination.

## Conclusion

To enable an effective cybersecurity strategy for SME’s and alike, government and private sector finance initiatives are key. This includes awareness and training for management, with oversight and additional support for staff to incorporate ML and AI into the workplace more effectively. Intrusion, detection and prevention policy from SME to government level can then flourish in promoting and sustaining the full benefits and protections of cybersecurity from cyber-criminality. However, for global data security coverage, the concept in itself is interpreted differently as the legal, ethical and consensual implementation challenges remain formidable as a result. Acquiring personal data from regional divisions to aide authorities in resource strategy at this scale, requires trust in institutions and technologies to be fully beneficial to all.

Accountability and transparency efforts also require the continual assessment of legal frameworks, systems and outcomes, with generous investment from public and private sectors. Public awareness, perception and confidence levels in the justice system through transparency and education, with focus to include mental illness and minority group engagement policies, can benefit societies substantially. The earlier proposed framework from research, demonstrates a robust and complex strategy, however looking to the future, BC network latency present real-time challenges to assist SME technology adoption. Increasing digitalisation and decentralisation leads to diverse communications, hence creating a wider array of participants to collaborate and share. However, these digital systems are not mature in terms of security and inevitably create attack space for attackers.

In this review paper, we highlighted several security problems that arise in digital systems, computation data and associated trust mechanisms. These challenges have resulted in evolution of technical solutions. Current solutions are so diverse that range from preliminary in small organisations to the state-of-the-art in mega-organisations. The cyber landscape is likely to change even further that necessitates robust solutions. This paper also brings in research from different collaborators with the potential to identify the challenges and move in the direction of designing novel solutions. This we believe as a result, will enhance and lead to secure cyber systems which achieve data security comprehensiveness.
